# Bridging the Past and the Future: Why Age Matters in Behavioral Health Training

**DOI:** 10.1093/geroni/igaa057.3052

**Published:** 2020-12-16

**Authors:** Rebecca Allen, Keisha Carden

## Abstract

This symposium presents data from three applied clinical research projects that involve intergenerational interaction as one component of effective treatment. The first paper describes learner outcomes in an intergenerational art therapy and reminiscence program provided in an adult day service facility. Results show that, in comparison with students in a didactic psychology of aging course or an introductory psychology course, learners in the experiential learning course demonstrated increased empathy, as well as better attitudes toward and increased interest in working with persons with dementia (PWD). The second paper focuses on observed outcomes for older PWD participants in this art therapy and reminiscence program, showing that intergenerational communication engagements exceed engagements with art. Mixed method data across time indicated that PWD benefitted from the treatment, facilitated by undergraduate student learners. The third paper focuses on cultural humility and the importance of racial diversity in providers conducting behavioral health screening in an integrated geriatric primary care clinic. Training issues and behavioral health outcomes regarding assessment of cognitive status, cultural mistrust, and test validity are considered. The fourth and final paper considers how intergenerational dynamics facilitated group cohesion and allowed for increased normalization of common challenges in mindfulness practice. Training issues for graduate student therapists are described. Consideration of level of behavioral health integration in each site, treatment efficacy, and the impact of intergenerational relationships are the foci of discussion. This symposium will show why age matters in behavioral health training.

